# Integrated analysis of multiple microarray studies to establish differential diagnostic models of Crohn’s disease and ulcerative colitis based on a metalloproteinase-associated module

**DOI:** 10.3389/fimmu.2022.1022850

**Published:** 2022-11-21

**Authors:** Jiang Deng, Ning Zhao, Li-ping Lv, Ping Ma, Yang-yang Zhang, Jin-bo Xu, Xi-peng Zhou, Zi-an Chen, Yan-yu Zhang

**Affiliations:** ^1^ Institute of Health Service and Transfusion Medicine, Beijing, China; ^2^ Beijing Key Laboratory of Blood Safety and Supply Technologies, Beijing, China; ^3^ Department of Gastroenterology, The Second Hospital of Hebei Medical University, Shijiazhuang, Hebei, China; ^4^ Hebei Key Laboratory of Gastroenterology, Hebei Institute of Gastroenterology, Hebei Clinical Research Center for Digestive Disease, Shijiazhuang, Hebei, China

**Keywords:** crohn’s disease, ulcerative colitis, robust rank aggregation, microarray, metalloproteinases, differential diagnostic models

## Abstract

**Background:**

The ulcerative colitis (UC) and Crohn’s disease (CD) subtypes of inflammatory bowel disease (IBD) are autoimmune diseases influenced by multiple complex factors. The clinical treatment strategies for UC and CD often differ, indicating the importance of improving their discrimination.

**Methods:**

Two methods, robust rank aggregation (RRA) analysis and merging and intersection, were applied to integrate data from multiple IBD cohorts, and the identified differentially expressed genes (DEGs) were used to establish a protein−protein interaction (PPI) network. Molecular complex detection (MCODE) was used to identify important gene sets. Two differential diagnostic models to distinguish CD and UC were established *via* a least absolute shrinkage and selection operator (LASSO) logistic regression, and model evaluation was performed in both the training and testing groups, including receiver operating characteristic (ROC) curves, calibration plots and decision curve analysis (DCA). The potential value of MMP-associated genes was further verified using different IBD cohorts and clinical samples.

**Results:**

Four datasets (GSE75214, GSE10616, GSE36807, and GSE9686) were included in the analysis. Both data integration methods indicated that the activation of the MMP-associated module was significantly elevated in UC. Two LASSO models based on continuous variable (Model_1) and binary variable (Model_2) MMP-associated genes were established to discriminate CD and UC. The results showed that Model_1 exhibited good discrimination in the training and testing groups. The calibration analysis and DCA showed that Model_1 exhibited good performance in the training group but failed in the testing group. Model_2 exhibited good discrimination, calibration and DCA results in the training and testing groups and exhibited greater diagnostic value. The effects of Model_1 and Model_2 were further verified in a new IBD cohort of GSE179285. The MMP genes exhibited high value as biomarkers for the discrimination of IBD patients using published cohort and immunohistochemistry (IHC) staining data. The MMP-associated gene levels were statistically significantly positively correlated with the levels of the differentially expressed cell types, indicating their potential value in differential diagnosis. The single-cell analysis confirmed that the expression of ANXA1 in UC was higher than that in CD.

**Conclusion:**

MMP-associated modules are the main differential gene sets between CD and UC. The established Model_2 overcomes batch differences and has good clinical applicability. Subsequent in-depth research investigating how MMPs are involved in the development of different IBD subtypes is necessary.

## Introduction

Inflammatory bowel disease (IBD) leads to chronic intestinal inflammation, is associated with significant morbidity, and results from the intersection of genetic and environmental factors that influence immune responses (1). Crohn’s disease (CD) and ulcerative colitis (UC) are the two main types of inflammatory bowel disease. Despite certain common pathological and clinical characteristics, CD and UC have several differences that indicate they are two distinct disease types. CD is characterized by fissuring ulceration, granulomatous inflammation and submucosal fibrosis. However, the characteristic histological findings in UC include crypt distortion, lymphocyte infiltration and chronic inflammation of the rectum, which is usually limited to the lamina propria (2, 3). Clinically, the diagnosis of IBD is usually established by a collective assessment of the clinical presentation and endoscopic, histopathological, radiographic and laboratory findings (4, 5).

An objective and clear discrimination between CD and UC diagnoses in patients with IBD colitis is currently vital for a tailored treatment plan since each disease involves different therapeutic and coping mechanisms (6–9). However, the differential diagnosis of these subtypes remains a remarkable clinical challenge since there is no single diagnostic gold standard for either UC or Crohn’s colitis (6–11). According to the public literature, approximately 5% to 15% of patients do not meet the strict criteria for either UC or CD (12–14), and the diagnoses of up to 14% of patients change over time (15–19). Therefore, the diagnostic assessment of IBD is often challenging; discriminating between CD and UC can be particularly challenging in patients in whom the inflammatory lesions are limited to the colon (20).

In recent years, with the development of high-throughput microarray technology, several studies have reported miRNAs as candidate biomarkers in IBD diagnostic assessment (21); however, few studies reported that mRNAs can be directly used for the differential diagnosis of CD and UC. Metalloproteinases (MMPs) belong to a large group of zinc-dependent proteolytic enzymes involved in degrading and remodeling the extracellular matrix (ECM) by cleaving specific components (22). In this study, we integrated data obtained from multiple IBD cohorts using two methods to identify important functional gene sets that differed between CD and UC and identified that the activation of the MMP-associated module was significantly elevated in UC. Furthermore, using different analytical methods, we established two differential diagnostic models to distinguish CD and UC *via* a least absolute shrinkage and selection operator (LASSO) logistic regression and further verified the models’ efficiency in several different cohorts. Finally, we explored the roles of these genes in immunity during the progression of UC, providing evidence that the expression of MMP-associated genes is correlated with the presence of multiple immune cell types. Thus, our diagnostic models provide promising diagnostic tools that might soon improve clinical practice.

## Materials and methods

### Search strategy for microarray datasets

**Figure 1:**
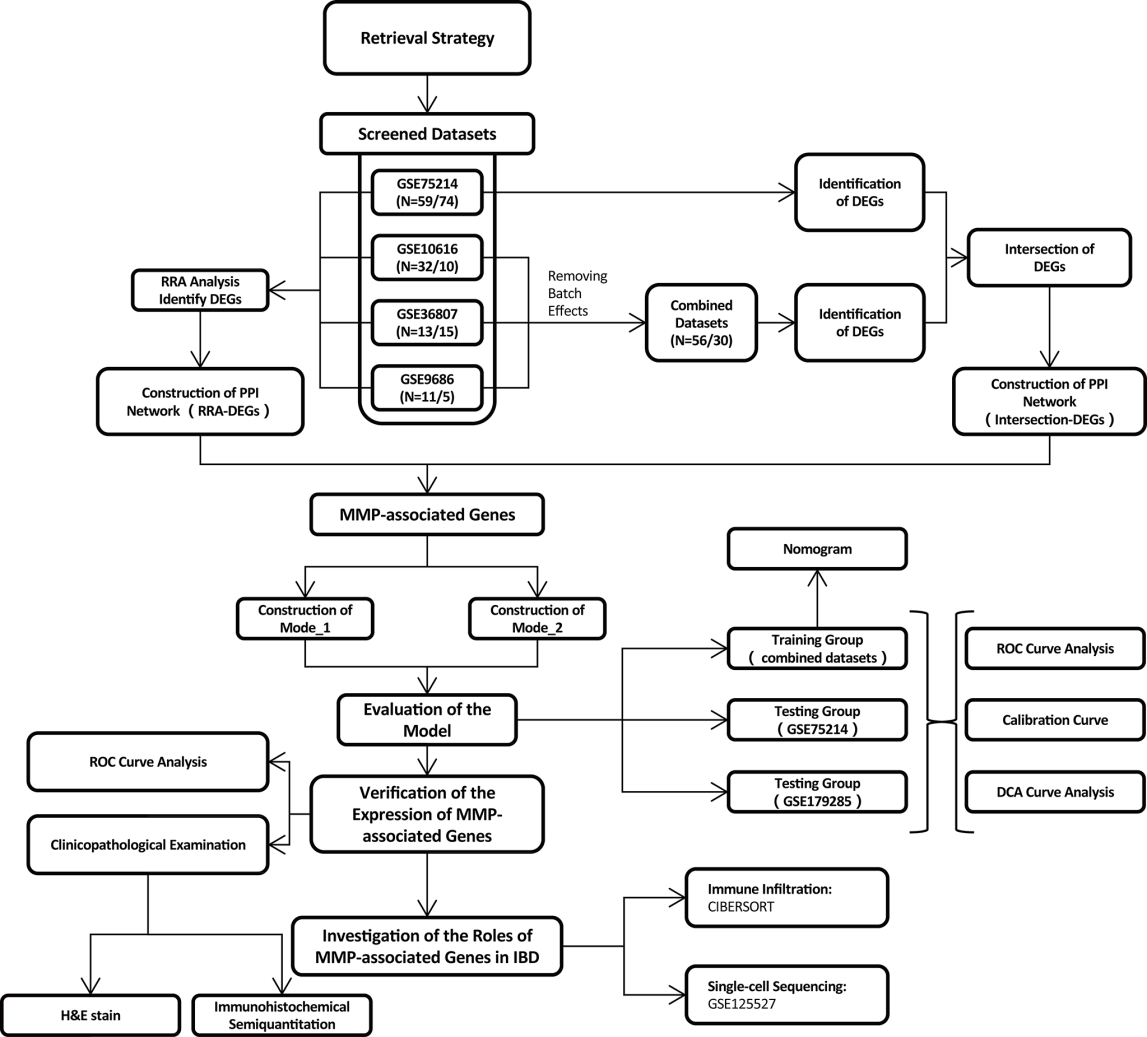
Flowchart of the overall study design.

In total, 139 datasets were collected from the Gene Expression Omnibus (GEO) database (https://www.ncbi.nlm.nih.gov/geo/) by systematic retrieval using the following keywords: (“Inflammatory Bowel Diseases”[MeSH Terms] OR Inflammatory Bowel Diseases [All Fields]) AND “Homo sapiens”[porgn] AND (“Expression profiling by array”[Filter] AND (“2008/01/01”[PDAT]: “2021/01/01”[PDAT]). The inclusion criteria were (1) a sample size > 15 (2); the inclusion of both CD and UC samples (3); sample sources of “ileum/colon”; and (4) available gene annotation information. To further verify the effectiveness of the models, a newly published IBD cohort, GSE179285, was used to evaluate the two models simultaneously. Another dataset, GSE125527, generated from single-cell sequencing was analyzed to investigate the role of MMP-associated genes in different cell clusters. The flow of the experimental design and data analysis is shown in **Figure 1**. The detailed information of the characteristics of the included datasets is shown in **Table 1**.

**Table 1:** Characteristic of the included microarray datasets.

GSE ID	Participants (CD/UC)	Analysis type	Platform	Year	Tissues	Sex (Male: Female)	Included in the RRA analysis	Network address
GSE75214	59/74	Array	GPL6244	2017	8 Colon and 51 Ileum in CD/74 Colon in UC	Not determined	Yes	https://www.ncbi.nlm.nih.gov/geo/query/acc.cgi?acc=GSE75214
GSE10616	32/10	Array	GPL5760 (identical to GPL570)	2009	14 Colon and 18 Ileo-colonic in CD/10 Colon in UC	Not determined	Yes	https://www.ncbi.nlm.nih.gov/geo/query/acc.cgi?acc=GSE10616
GSE36807	13/15	Array	GPL570	2013	Not determined	9:4 in CD/8:7 in UC	Yes	https://www.ncbi.nlm.nih.gov/geo/query/acc.cgi?acc=GSE36807
GSE9686	11/5	Array	GPL5760 (identical to GPL570)	2008	11 Colon in CD/5 Colon in UC	Not determined	Yes	https://www.ncbi.nlm.hih.gov/geo/query/acc.cgi?acc=GSE9686
GSE179285	37/23	Array	GPL6480	2021	14 Colon and 33 Ileo in CD/23 Colon in UC	Not determined	No	https://www.ncbi.nlm.nih.gov/geo/query/acc.cgi?acc=GSE179285
GSE125527	7/7	Single-cell Sequencing	GPL20301	2020	7 Rectum in CD/7 Rectum in UC	Not determined	No	https://www.ncbi.nlm.nih.gov/geo/query/acc.cgi?acc=GSE125527

### Robust rank aggregation analysis and identification of differentially expressed genes in the integrated cohort

Using RRA, all genes in each dataset were sorted and ranked based on their log fold-change (logFC) values using the limma package. The DEGs were then ranked using the ranked list and aggregated using the RRA package in R software. Using this method, an adjusted P value determines the likelihood that DEGs will be identified in the datasets with highly ranked genes. LogFC values > 0.7 and adjusted P values < 0.05 were set as the criteria for identifying DEGs.

### Identification of DEGs by merging and intersection

To increase the sample size of the IBD cohort, three datasets (GSE10616, GSE36807, and GSE9686) from the same platform were merged and named Combined Datasets. The ComBat function was used to remove batch effects using the SVA package. Then, the DEGs were identified in the Combined Datasets and GSE75214 with the criteria of LogFC values > 0.6 and adjusted P values < 0.1. The final DEGs were identified by considering the intersection of the DEGs between the two IBD cohorts.

### Functional and pathway enrichment analyses

We performed a Gene Ontology biological process (GO-BP) analysis and Kyoto Encyclopedia of Genes and Genomes (KEGG) analysis of the DEGs identified by the RRA analysis using the limma and clusterProfiler packages. The enrichment analysis of the DEGs was performed with the criteria of adjusted P values of < 0.05 (23).

### Establishment of a PPI network and MCODE analysis

Using the DEGs obtained by the RRA analysis/intersection, a PPI network was established using the STRING website (https://cn.string-db.org/), with a parameter of confidence of > 0.4. Visualization of the PPI network was performed by Cytoscape (v3.7.2), and molecular complex detection (MCODE) (a plug-in in Cytoscape) was used to identify the functional modules (24).

### Establishment of model_1 *via* LASSO logistic regression

For the logistic regression, to achieve a high performance, LASSO was applied to reduce the dimensions of the analysis. The candidate genes were collected from the combination of two MMP-associated modules identified in the PPI network, including MMP3, MMP1, MMP12, PLAU, MMP9, CXCL1, MMP10, PTGS2, TIMP1, MMP7, CXCL13, S100A12, S100A8, S100A9, and ANXA1. The combined datasets were set as the training group, while GSE75214 was used as the testing group to verify the effect of the model.

The LASSO procedure involved performing a logistic regression with an L1 regularization penalty, which has the effect of shrinking the regression weights of the least predictive features to 0. To determine the coefficients of optimal penalty, we performed tenfold cross-validation, and binomial deviation was used as a performance measure. Therefore, the function cv.glmnet was used with the following parameters: alpha = 1, nfolds = 10, and type.measure = “deviance”. To obtain parsimonious models, the largest lambda, which was within one standard error of the minimum training deviance as recommended, was used to establish the final model.

### Establishment of model_2 *via* LASSO logistic regression

To correct the model application problems caused by the batch differences across different platforms, we used another method to generate the model. A binary variable translation was performed with 15 candidate MMP-associated genes to obtain a new index for each MMP-associated gene in each sample. For genes with increased expression in UC, the binary variable of MMP-associated genes was assigned a value of 1 if the expression value of a gene was greater than the median of the expression value of that gene in all samples; otherwise, the index was defined as 0. For genes with increased expression in CD, the binary variable of MMP-associated genes was assigned a value of 1 if the expression value of a gene was less than the median of the expression value of that gene in all samples; otherwise, the index was defined as 0. Therefore, the expression values of 15 genes were converted from continuous variables into binary variables. Similar to the method used to establish Model_1, the combined datasets were set as the training group, while GSE75214 was used as the testing group to verify the effect of the model.

Model_2 was generated using a different method than model_1. To determine the coefficients of optimal penalty, we performed 8-fold cross-validation, and the area under the receiver operator characteristics (ROC) curve was used as a performance measure. Thus, the function cv.glmnet in the package glmnet v2.0-16 was used with the following parameters: alpha = 1, nfolds = 8, and type.measure = “auc”. To obtain parsimonious models, the largest lambda, which was within one standard error of the maximal training AUC as recommended, was used to generate the final model.

### Evaluation of the differential diagnostic models

The models were developed and strictly validated according to the Transparent Reporting of a multivariable prediction model for Individual Prognosis Or Diagnosis (TRIPOD) guidelines (25). Specifically, we used ROC analyses and the AUC to assess the discriminatory ability of the model in discriminating CD from UC cases. Calibration plots were drawn to assess the goodness of fit of each model. A decision curve analysis (DCA) was performed to assess the clinical net benefit of each model and compare the model with the use of all strategies and random chance. The equations used in the final models are presented as nomograms. Importantly, the ROC analysis, calibration plot assessments and DCA were performed in both the training and testing groups, while the nomogram was illustrated only in the training group according to the TRIPOD guidelines. All analyses were conducted using R version 4.1.3.

### Evaluation of immunohistochemical staining

To validate the results of the genetic analysis at the transcriptional level, human intestinal mucosal tissues were collected from the Department of Gastroenterology at the Second Hospital of Hebei Medical University between 2020-2021. The histopathologic diagnosis was made by two pathologists, and the sample set included 15 CD samples and 23 UC samples.

The collected intestinal mucosa of the patients were fixed with 4% paraformaldehyde (PFA) and embedded in paraffin. IHC staining was performed as previously described (26). The following antibodies were used: MMP7 (A20701), ANXA1 (A1118), MMP10 (A3033), HRP-labeled goat anti-rabbit antibody (AS014; 1:200 dilution, all from ABclonal, Wuhan, China), CXCL13 (bs-2553R), and CXCL1 (bs-10234R, all from Bioss, Beijing, China; 1:200 dilution).

The samples were scored by two trained pathologists according to the percentage contribution of the high positive, positive, low positive, and negative samples. The immunoreactive score (IRS) was evaluated as follows: 4, high positive; 3, positive; 2, low positive; and 1 negative (27).

### Landscape of immune cell infiltration

To evaluate immune cell infiltration, CIBERSORT was used to quantify 22 tumor-infiltrating immune cell subgroups in the CD and UC groups in both the Combined Datasets and the GSE75214 dataset. Because the MMP-associated genes exhibited a higher level in UC in this study, the relationships between the expression of immune cell subgroups and MMP-associated genes in UC were further examined by a Spearman correlation analysis.

### Single-cell sequencing analysis

The single-cell sequencing analysis was based on public data (GSE125527, including 7 CD and 7 UC patients) downloaded from the GEO website and analyzed *via* R software according to the following steps: 1) the “Seurat” package was adopted to convert 10× scRNA-seq data as a Seurat object; 2) the “FindVariableFeatures” function was adopted to filter the top 2000 highly variable genes; 3) a principal component analysis (PCA) was performed based on the 2000 genes, and the data from different samples were further merged *via* harmony integration; 4) a clustering analysis was performed to find subtypes, and uniform manifold approximation and projection (UMAP) was used for dimensionality reduction and cluster identification.

### Statistical analysis

All statistical tests were implemented using R software 4.1.3. A Wilcoxon test was used to analyze the significance of the differences between the groups. Spearman’s correlation test was used to determine the correlation between the variables. Statistically significant results were defined as those with P values < 0.05.

## Results

### Characteristics of the included microarray datasets

After conducting a systematic search based on the inclusion criteria described in the Materials and Methods, in total, four microarray datasets of IBD patients were retrieved from the GEO database, including GSE75214 (N=59/74, x/y=the number of CD/UC included in the research) (28), GSE10616 (N=32/10) (29), GSE36807 (N=13/15) (30), and GSE9686 (N=11/5) (31). Therefore, in total, 115 cases of CD and 104 cases of UC were finally included in the analysis.

### Identification of DEGs by an RRA analysis and functional enrichment analysis

Considering that the collected data were generated from different microarray platforms, combining the datasets by using direct merging inevitably led to erroneous conclusions caused by bias. Therefore, the RRA method was first applied to identify DEGs in the four GEO datasets.

**Figure 2:**
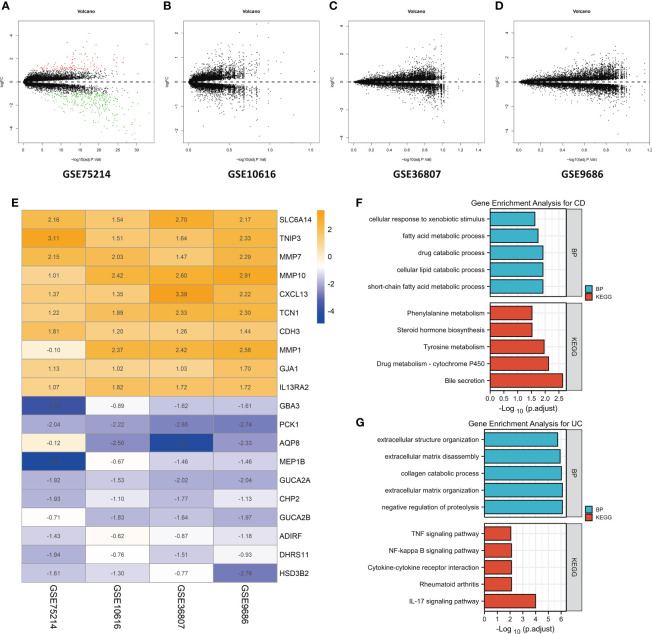
Identification of DEGs by an RRA analysis and functional enrichment analysis. Volcano plots of the DEG distributions in the GSE75214 **(A)**, GSE10616 **(B)**, GSE36807 **(C)**, and GSE9686 **(D)** datasets. Upregulated genes are indicated by red points, while downregulated genes are indicated by green points. Genes with no significant differences in levels are indicated by black points. **(E)** Heatmap of the expression data of the top 10 DEGs identified in the RRA. The genes upregulated in CD are indicated in blue, and those upregulated in UC are indicated in orange. Functional enrichment analysis of DEGs, including **(F)** a GO-BP analysis and **(G)** a KEGG pathway analysis.

First, the datasets were standardized to correct batch differences within the datasets, and the results showed that the homogeneity of the data met the requirements (**Supplementary Figure 1**). A volcano map of each dataset was produced using the Limma package in R software, and the DEGs are indicated as green and red points in **Figures 2A-D**. The results showed that the DEGs greatly differed across the different datasets. In the GSE75214 dataset, many DEGs were identified, while few DEGs were identified in the GSE10616, GSE36807, and GSE9686 datasets as indicated in the figures, suggesting that the data collected from the cohorts across different sources were heterogeneous.

After the RRA analysis, in total, 141 DEGs (86 overexpressed in UC and 55 overexpressed in CD) were finally identified, and a heatmap of the expression data of the top 20 DEGs is shown in **Figure 2E**. The top 10 significant genes that were aberrantly expressed in UC included five genes [SLC6A14 (P = 2.78E-08), TNIP3 (P = 1.03E-07), MMP7 (P = 4.45E-07), MMP10 (P = 1.68E-06), and CXCL13 (P = 2.09E-06)], and five genes were overexpressed in CD [GBA3 (P = 7.48E-07), PCK1 (P = 3.27E-06), AQP8 (P = 8.98E-06), MEP1B (P = 1.82E-05), and GUCA2A (P = 3.48E-05)]. The overall results of the RRA analysis are listed in **Supplementary Table 1**.

The DEGs were subjected to a GO-BP analysis and KEGG analysis, and the top five results are listed in **Figures 2F, G**. The results showed that the short-chain fatty acid metabolic process, cellular lipid catabolic process, drug catabolic process, fatty acid metabolic process and cellular response to xenobiotic stimulus were the top five enriched BPs, while bile secretion, drug metabolism - cytochrome P450, tyrosine metabolism, steroid hormone biosynthesis, and phenylalanine metabolism were the top five enriched KEGG pathways in CD. In the UC samples, negative regulation of proteolysis, extracellular matrix organization, collagen catabolic process, extracellular matrix disassembly, and extracellular structure organization were the top five enriched BPs, while the IL-17 signaling pathway, rheumatoid arthritis, cytokine−cytokine receptor interaction, NF-kappa B signaling pathway and TNF signaling pathway were the top five enriched KEGG pathways. The detailed results are listed in **Supplementary Table 2**.

### Identification of DEGs by merging and intersection and a functional enrichment analysis

**Figure 3:**
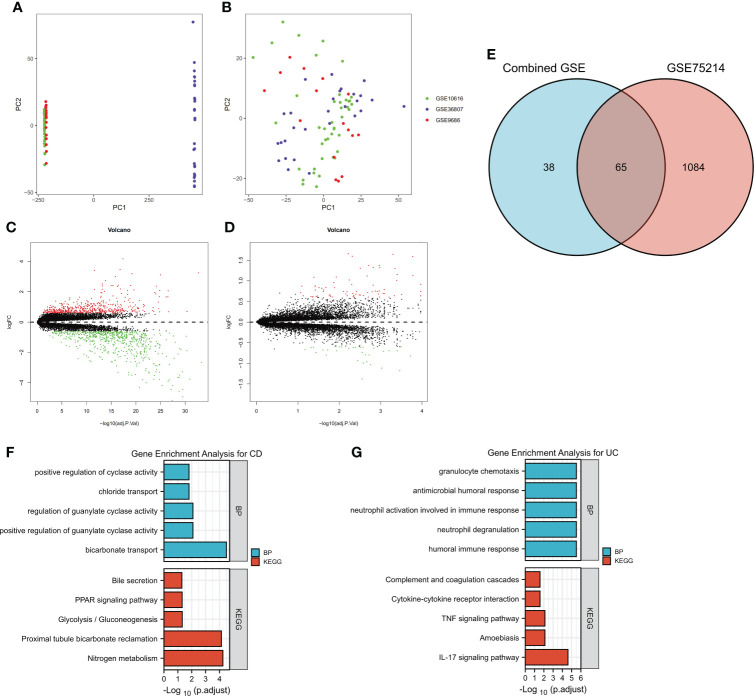
Identification of DEGs by merging and intersection and a functional enrichment analysis. The GSE10616, GSE36807 and GSE9686 datasets were merged, and batch effects were further removed. PCA plots of different datasets are illustrated before **(A)** and after **(B)** batch effects were removed. Volcano plots of the DEG distributions in the GSE75214 dataset **(C)** and Combined Datasets **(D)**. **(E)** The intersection of DEGs in the GSE75214 dataset and Combined Datasets is displayed as a Venn diagram. Functional enrichment analysis of DEGs, including **(F)** a GO-BP analysis and **(G)** a KEGG pathway analysis.

To identify the DEGs more comprehensively, we adopted another method. Because the data in the GSE10616, GSE36807 and GSE9686 datasets were collected using the same platform, combined datasets (named Combined Datasets, N=56/30) were generated by merging the GSE10616, GSE36807 and GSE9686 datasets by removing batch effects *via* the SVA package in R software. The distribution of the data before and after correction was tested by a principal component analysis (PCA) dimensionality reduction (**Figures 3A, B**). The results showed that the distribution of each dataset was quite different before the batch correction, while the distribution of the data after correction overlapped well.

The differentially expressed genes in the GSE75214 dataset and Combined Datasets were analyzed (**Figures 3C, D**). The final DEGs were determined by considering the intersection of the differentially expressed genes, and in total, 65 DEGs were identified as listed in **Figure 3E** and **Supplementary Table 3**.

**Figure 4:**
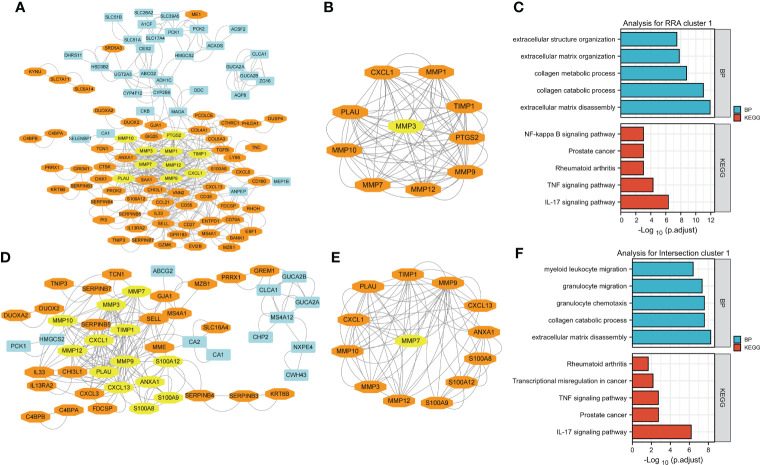
Visualization and module identification using two different PPI networks. **(A)** RRA-DEGs were mapped using Cytoscape software (genes mainly expressed in CD are indicated in blue, and genes mainly expressed in UC are indicated in orange). Using the MCODE plug-in, the module with the highest scores was identified (indicated in yellow). **(B)** The identified module comprised MMP1, MMP12, PLAU, MMP9, CXCL1, MMP10, PTGS2, TIMP1, and MMP7 (indicated in orange), with MMP3 as the seed gene (indicated in yellow). Functional enrichment analyses of the genes in the MMP-associated module were performed **(C)**. **(D)** The intersection-DEGs were mapped using Cytoscape software (genes mainly expressed in CD are indicated in blue, and genes mainly expressed in UC are indicated in orange). Using the MCODE plug-in, the module with the highest scores was identified (indicated in yellow). **(E)** The identified module comprised MMP12, MMP10, MMP3, MMP9, TIMP1, CXCL1, PLAU, S100A9, CXCL13, S100A8, ANXA1 and S100A12 (indicated in orange), with MMP7 as the seed gene (indicated in yellow). Functional enrichment analyses of the genes in the MMP-associated module were performed **(F)**.

The DEGs, including 45 overexpressed in UC and 20 overexpressed in CD, were subjected to a GO-BP analysis and KEGG analysis, and the top five results are listed in **Figures 4F, G**. The results showed that bicarbonate transport, positive regulation of guanylate cyclase activity, regulation of guanylate cyclase activity, chloride transport, and positive regulation of cyclase activity were the top five enriched BPs, while nitrogen metabolism, proximal tubule bicarbonate reclamation, glycolysis/gluconeogenesis, the PPAR signaling pathway, and bile secretion were the top five enriched KEGG pathways in the CD samples. In the UC samples, humoral immune response, neutrophil degranulation, neutrophil activation involved in immune response, antimicrobial humoral response, and granulocyte chemotaxis were the top five enriched BPs, while the IL-17 signaling pathway, amoebiasis, TNF signaling pathway, cytokine−cytokine receptor interaction, and complement and coagulation cascades were the top five enriched KEGG pathways. The detailed results are listed in **Supplementary Table 4**. Notably, although we used two different methods to identify the DEGs, the results showed that the identified enriched signaling pathways were highly similar, including the IL-17 signaling pathway, TNF signaling pathway, and cytokine−cytokine receptor interaction in UC.

### MMP-associated module is the most important network module in both PPI networks

For clarity, we named the DEGs identified in the RRA analysis RRA-DEGs, and the DEGs identified from the intersection of the GSE75214 dataset and Combined DataSets were named Intersection-DEGs. Two PPI networks based on the RRA-DEGs and intersection-DEGs were generated to better explore the differences in complex regulatory mechanisms between CD and UC using the STRING website, and the results were visualized by Cytoscape software.

The network generated by using the RRA-DEGs is shown in **Figure 4A** and includes 101 nodes and 464 edges. The Molecular Complex Detection (MCODE) function module is a commonly used module in the establishment of PPI networks. This module can be used to identify important subnetworks and genes in a large PPI network according to the relationship between the edges and nodes to facilitate the following analysis. Through a MCODE analysis, we observed that the most important subnetworks mainly involved MMP family genes, including MMP1, MMP12, PLAU, MMP9, CXCL1, MMP10, PTGS2, TIMP1, and MMP7, with MMP3 as the seed gene (**Figure 4B**). Through a gene enrichment analysis, we observed that extracellular matrix disassembly, collagen catabolic process, collagen metabolic process, extracellular matrix organization, and extracellular structure organization were the top five enriched BPs, while the IL-17 signaling pathway, TNF signaling pathway, rheumatoid arthritis, prostate cancer, and NF-kappa B signaling pathway were the top five enriched KEGG pathways (**Figure 4C** and details in **Supplementary Table 5**).

Then, we used the intersection-DEGs to generate another PPI network. The results are shown in **Figure 4D**, which shows 49 nodes and 190 edges. As before, the MCOD plug-in was used to identify important subnetworks and key genes. Although fewer DEGs were used to generate the PPI network in this analysis, the key subnetworks finally identified still mainly involved the MMP family, including MMP12, MMP10, MMP3, MMP9, TIMP1, CXCL1, PLAU, S100A9, CXCL13, S100A8, ANXA1 and S100A12, with MMP7 as the seed gene (**Figure 4E**). Through a gene enrichment analysis, we found that extracellular matrix disassembly, collagen catabolic process, granulocyte chemotaxis, granulocyte migration, and myeloid leukocyte migration were the top five enriched BPs, while the IL-17 signaling pathway, prostate cancer, TNF signaling pathway, transcriptional misregulation in cancer, and rheumatoid arthritis were the top five enriched KEGG pathways (**Figure 4F** and details in **Supplementary Table 5**).

Notably, the results of both PPI networks independently identified the MMP family as a pivotal module, and the results of the gene functional enrichment analysis of both MMP modules were quite similar to those obtained by analyzing all DEGs in the RRA/intersection analysis as shown in **Figures 2F, G** and **Figures 3F, G**; this finding indicates that the module of the MMP family is the main differential gene set between CD and UC.

### Establishment of model_1 based on the MMP-associated module *via* a LASSO logistic regression

**Figure 5:**
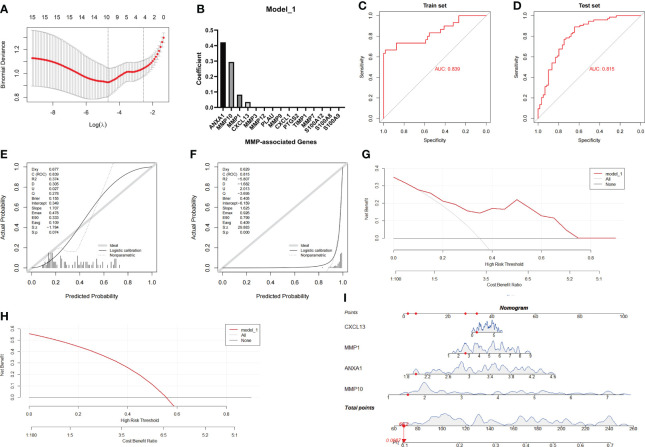
Establishment and evaluation of Model_1 with the MMP-associated module. **(A)** Selection of the optimal parameter (lambda) in the LASSO model. **(B)** Plot of coefficients obtained by LASSO. ROC curve **(C, D)**, calibration curves **(E, F)**, and decision curve analysis (DCA) **(G, H)** of the training and testing cohort models. **(I)** Nomogram of Model_1 for the predictive value of CD/UC is illustrated.

Based on the above results, we speculated that the MMP family module was the main differential gene set between UC and CD. Therefore, the genes in two MMP subnetworks were merged and served as candidates for establishing the LASSO logistic regression model. The combined datasets were set as the training group, while the GSE75214 dataset was set as the testing group. After the model reached the minimum+1 standard error lambda, an MMP-related signature with 4 components was built discriminate between CD and UC (**Figure 5A**). The diagnostic score was defined as -3.7469+[expression level of ANXA1 × (0.4213)]+[expression level of MMP10 × (0.2944)]+[expression level of MMP1 × (0.0826)]+[expression level of CXCL13 × (0.0351)], and the optimal features and their coefficient values are shown in **Figure 5B**. Notably, the diagnostic score was a scoring system used only for differential diagnosis, and the score was positively correlated with UC and negatively correlated with CD.

In the next investigation, we evaluated the diagnostic efficiency of Model_1 in discriminating between CD and UC. The methods of the evaluation strictly complied with the guidelines of the TRIPOD (Transparent Reporting of a multivariable prediction model for Individual Prognosis Or Diagnosis) Statement (25), including discrimination (ROC curve and AUC), calibration (calibration plot), decision curve analyses and a nomogram. The results of the ROC curve showed that the AUC achieved in the training group was 0.839, and that achieved in the testing group was 0.815 (**Figures 5C, D**). In addition, the calibration curve showed that the diagnostic score had a better prediction accuracy in the training group and poor calibration in the testing group (**Figures 5E, F**). Similarly, the DCA results showed that the diagnostic score in the training group served as a better indicator during clinical decision-making than that in the testing group (**Figures 5G, H**). The nomogram for the differential diagnosis of CD and UC is illustrated in **Figure 5I**. For example, for a patient with a CXCL13 value of 1.0416, MMP1 value of 2.6360, ANXA1 value of 1.9737, and MMP10 value of 1.5244, the predicted probability of the patient having UC was 0.0987, while the predicted probability of the patient having CD was 0.9013. According to a cutoff value of 0.5, the patient was identified as having CD based on Model_1.

### Establishment of model_2 based on the MMP family module *via* a LASSO logistic regression

Based on the poor calibration and clinical applicability of Model_1, a better method for establishing a diagnostic model was applied as described in the Methods and Material. The main difference was that the continuous variables representing gene expression were transformed into binary variables, leading to a reduction in distribution differences due to specific expression values.

**Figure 6:**
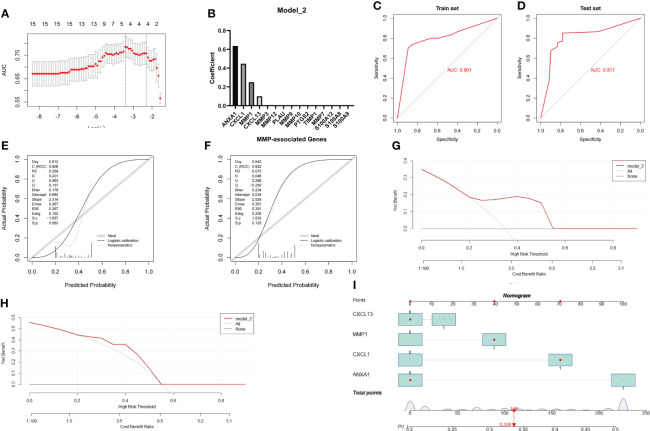
Establishment and evaluation of Model_2 with the MMP-associated module. **(A)** Selection of the optimal parameter (lambda) in the LASSO model. **(B)** Plot of coefficients obtained by LASSO. ROC curve **(C, D)**, calibration curves **(E, F)**, and decision curve analysis (DCA) **(G, H)** of the training and testing cohort models. **(I)** Nomogram of Model_2 for the predictive value of CD/UC is illustrated.

Similar to the method used to assess Model_1, the Combined Datasets were set as the training group, while the GSE75214 dataset was set as the testing group. After the model reached the minimum+1 standard error lambda, an MMP-related signature with 4 components was generated to discriminate between CD and UC (**Figure 6A**). The diagnostic score was defined as -1.3813+ [value of ANXA1×(0.6358)]+ [value of CXCL13×(0.1000)]+ [value of MMP1×(0.2507)]+ [value of CXCL1×(0.4478)], and the optimal features and their coefficient values are shown in **Figure 6B**.

Similar to the method used during the investigation of Model_1, we further evaluated the diagnostic efficiency of Model_2 in identifying CD and UC. The results of the ROC curve showed that the AUC achieved in the training group was 0.801, and that achieved in the testing group was 0.811 (**Figures 6C, D**). In addition, the calibration curve showed that the diagnostic score had a better prediction accuracy in both the training group and the testing group (**Figures 6E, F**). Similarly, the DCA results showed that the diagnostic score in both the training group and the testing group served as a better indicator in clinical decision-making (**Figures 6G, H**). The nomogram for the differential diagnosis of CD and UC is illustrated in **Figure 6I**. For example, for a patient with a CXCL13 value of 0, an MMP1 value of 1, an ANXA1 value of 0, and a CXCL1 value of 1, the predicted probability of a UC diagnosis was 0.336, while the predicted probability of a CD diagnosis was 0.664. According to a cutoff value of 0.5, the patient was identified as having CD based on Model_2. Overall, these results suggest that Model_2 based on the MMP-associated module has good prediction ability.

### Verification of the effectiveness of model_1 and model_2 in a new IBD cohort

Although the effects of Model_1 and Model_2 were tested in the above studies, considering that the GSE75214 cohort was included in the RRA analysis, strictly speaking, the GSE75214 cohort is not a complete test queue. Therefore, a recently published IBD cohort, GSE179285, which was not included in the RRA analysis, was used for a more rigorous evaluation of the effects of the two models.

**Figure 7:**
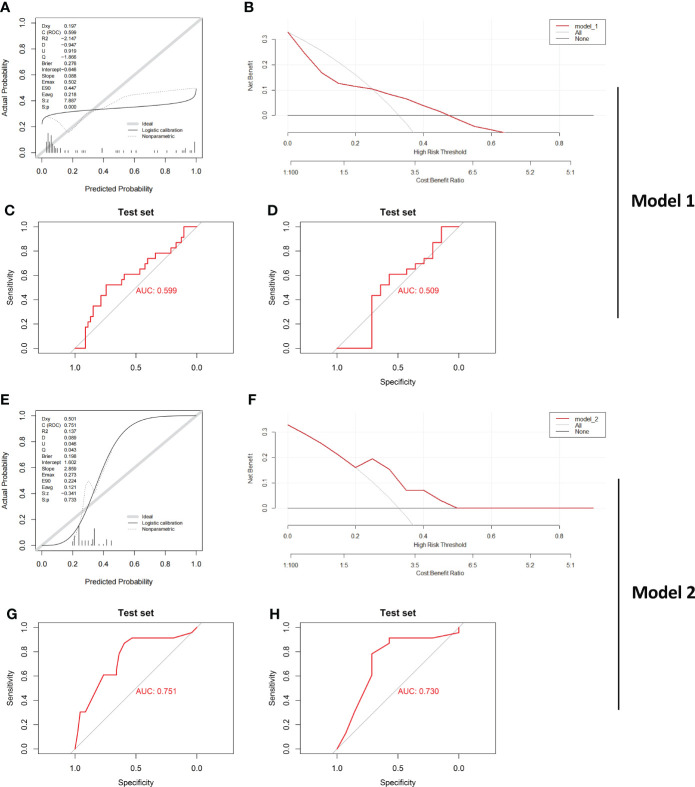
Verification of the effect of both Model_1 and Model_2 in a new IBD Cohort Calibration curves **(A)**, DCA curve **(B)** and ROC curve **(C)** testing the effect of Model_1 in GSE179285 are illustrated. CD and UC samples from colon tissue only were further examined by Model_1, and **(D)** ROC curve is shown. Calibration curves **(E)**, DCA curve **(F)** and ROC curve **(G)** testing the effect of Model_2 in GSE179285 are illustrated. CD and UC samples from colon tissue only were further examined by Model_2, and **(H)** ROC curve is shown. CD/UC=37/23 is the sample size in GSE179285, and CD/UC=14/23 is the sample size in GSE179285 including only colon tissue.

The evaluation results of Model_1 are shown in **Figures 7A-C**. The results show that the ROC curve, calibration curves and decision curve analysis of Model_1 are very poor likely because GSE179285 was generated from GPL6480, a platform different from the other queues. In addition, since the GSE179285 cohort contains a large number of colon tissue from CD cases, we used Model_1 to evaluate CD and UC in colon tissue only and showed that the differentiation of Model_1 was still poor (**Figure 7D**, AUC=0.509). In contrast, Model_2 still shows good ROC curves, calibration curves and decision curve analysis in the new cohort (**Figures 7E-G**), although the data were derived from a platform that the model never faced before. Furthermore, Model_2 still shows a good ROC curve between the UC and CD samples from only colon tissue, highlighting its great application value as a clinical diagnostic model (**Figure 7H**, AUC=0.730). As we know, the real challenge of differential diagnostic of two subtypes of IBD is between colonic dominant CD versus UC, which reflects the potential clinical value of the Model_2. We performed statistical analysis on CD and UC samples from colonic tissue in the former IBD cohort. The results showed that the model had an AUC of 0.674 in GSE75214 (CD/UC=8/74), 0.754 in GSE10616 (CD/UC=14/10), and 0.900 in GSE9686 (CD/UC=11/5, **Supplementary Figure 3**).

### Verification of MMP-associated genes in different IBD cohorts

**Figure 8:**
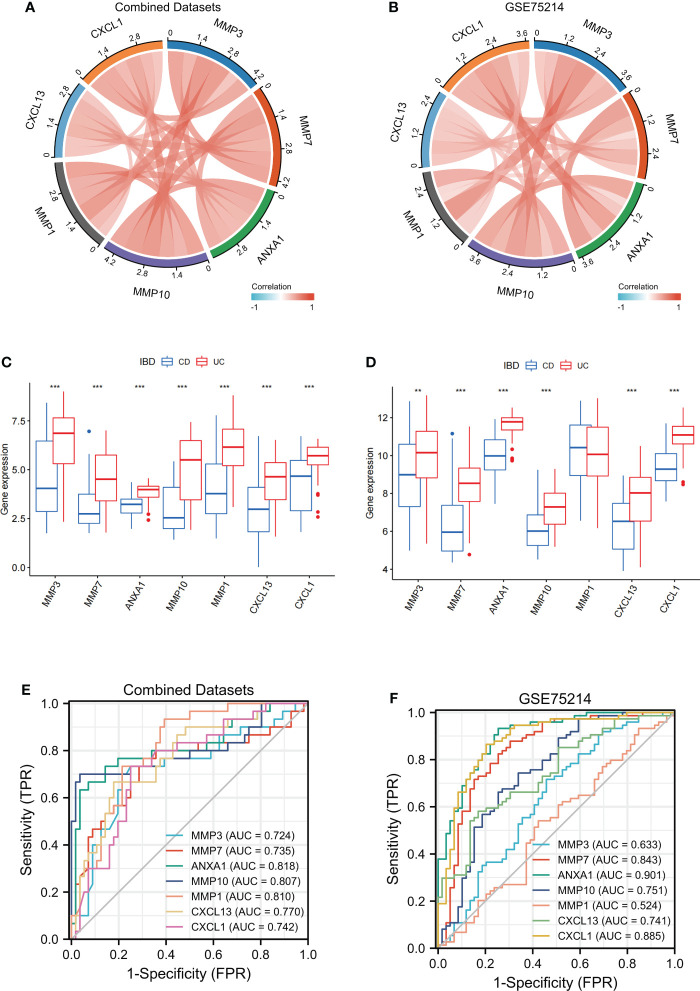
Verification of MMP-associated genes in different IBD cohorts The correlation between the levels of internal MMP-associated genes in both Combined Datasets **(A)** and the GSE75214 dataset **(B)** using a Spearman correlation analysis. The differences in MMP-associated genes between CD and UC were examined in both the Combined Datasets **(C)** and the GSE75214 dataset **(D)** using the limma package. ROC curves estimating the diagnostic performance of the MMP-associated genes ANXA1, MMP10, MMP1, CXCL13 and CXCL1 in the discrimination of IBD patients in the Combined Datasets **(E)** and GSE75214 **(F)**. ** indicates P<0.01, *** indicates P<0.001.

After establishing and evaluating the model, we explored the function and diagnostic value of MMP-associated genes, including the seed genes identified in the MCODE analysis (MMP3 and MMP7) and 5 genes assessed in Model_1 and Model_2 (ANXA1, MMP10, MMP1, CXCL13 and CXCL1). We first examined the correlation between the internal MMP-associated genes in both the Combined Datasets and the GSE75214 dataset using a Spearman correlation analysis, and the results are illustrated in **Figures 8A, B**. The results showed that multiple strong positive correlations were detected between the levels of the MMP-associated genes. In addition, the differences in the MMP-associated gene levels between CD and UC were examined, and the results showed statistically significant differences between the two groups in the expression of most MMP-associated genes, including MMP3, MMP7, ANXA1, MMP10, CXCL13 and CXCL1, except for MMP1 in the GSE75214 dataset (**Figures 8C, D**).

To further verify the value of the MMP-associated genes as diagnostic markers, we explored their ROC curves in different cohorts (**Figures 8E, F**). The results showed that the AUCs of ANXA1 and MMP10 were greater than 0.75, and those of MMP7, CXCL13 and CXCL1 were greater than 0.70 in the two cohorts, CD and UC. Therefore, these genes showed high value as biomarkers for the discrimination of IBD patients.

### Validation of the expression levels of MMP-associated genes in clinical samples

**Figure 9:**
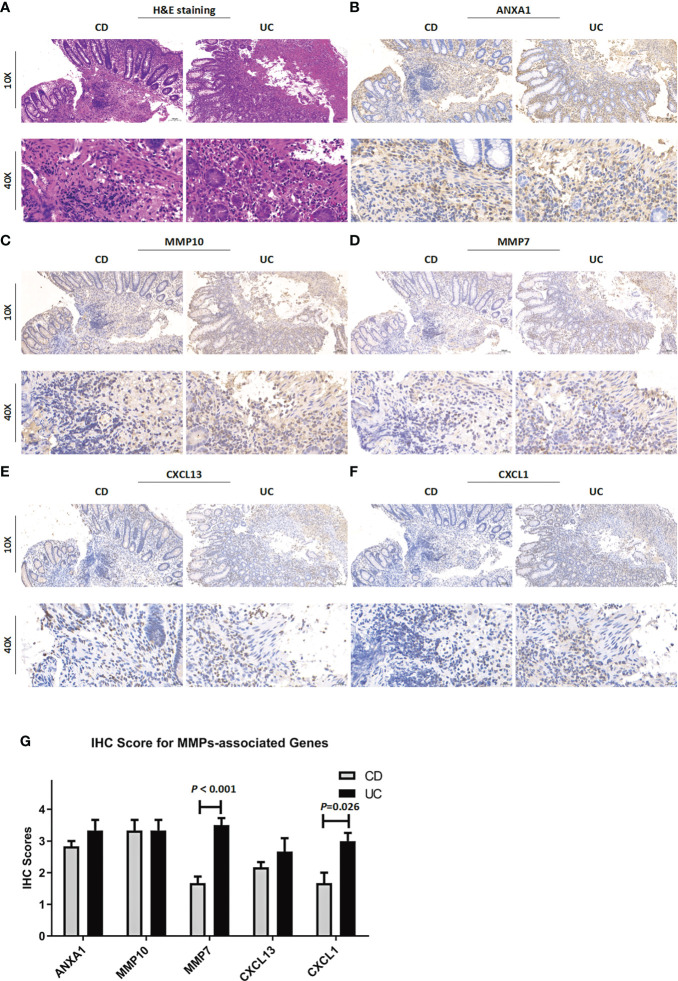
Clinicopathological examination of MMP-associated genes in tissue samples. **(A)** Representative images of H&E staining of samples from CD and UC patients. The IHC staining images are representative images of the expression levels of ANXA1 **(B)**, MMP10 **(C)**, MMP7 **(D)**, CXCL13 **(E)** and CXCL1 **(F)** in CD and UC samples. **(G)** IHC score difference analysis of MMP-associated genes in IBD patients. Statistical differences were analyzed by a Mann−Whitney U test.

To verify the reliability of the above results obtained from a public database, we collected intestinal mucosa samples from 38 patients (CD/UC =15/23) to test the expression levels of the MMP-associated genes. As shown in **Figure 9**, the expression levels of ANXA1, MMP10 and CXCL13 did not differ between CD and UC samples. However, MMP7 and CXCL1 showed higher levels in the UC patients than in the CD patients. After the scoring data were summarized and analyzed, the results showed that the differences in the MMP7 and CXCL1 levels between the CD and UC groups were statistically significant. However, due to the complexity of the etiology of IBD, we believe that IHC of a single index cannot accurately provide a reference for disease diagnosis, especially based on the judgment of a small clinical sample size.

### Analysis of the correlation between MMP-associated gene levels and the levels of infiltrating immune cells

**Figure 10:**
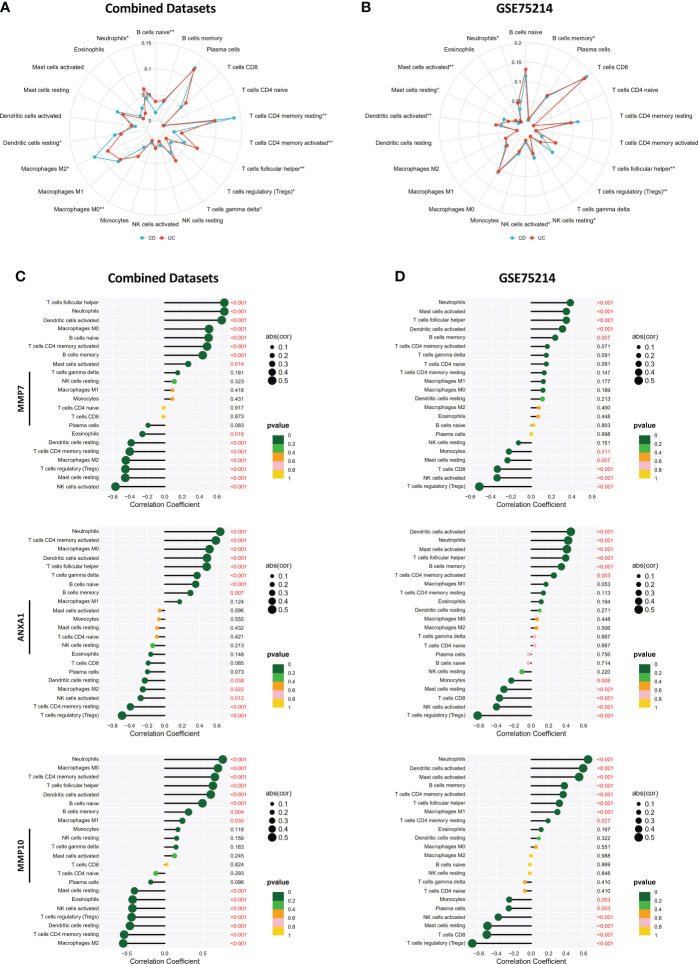
Correlations between MMP-associated gene levels and the levels of infiltrating immune cells determined by CIBERSORT. Differences in the levels of infiltrating immune cells between the CD and UC groups in the Combined Datasets **(A)** and the GSE75214 dataset **(B)**. Correlations between the MMP-associated gene levels and immune cell levels were further examined by a Spearman correlation analysis in the Combined Datasets **(C)** and the GSE75214 dataset **(D)**, and results with significant differences are shown (*p < 0.05, **p < 0.01). The results, including data regarding other MMP-associated genes, including CXCL13, MMP3, MMP1 and CXCL1, are illustrated in **Supplementary Figure 2**.

By performing CIBERSORT, we compared the infiltration levels of most immune cell populations between CD and UC patients in both the Combined Datasets and the GSE75214 dataset (**Figures 10A, B**). The results showed that several immune cell types, including neutrophils and humoral immune cells (naive B cells and follicular helper T cells), were more abundant in UC patients, while a higher level of Treg cells was observed in the CD group. In addition, the correlations between the MMP-associated gene levels and the levels of various immune cell populations were further examined by a Spearman correlation analysis (**Figures 10C, D**, and **Supplementary Figure 2**), and the results showed that the levels of all MMP-associated genes were statistically significantly positively correlated with the levels of the differentially expressed cell types, indicating their potential value as biomarkers during differential diagnosis.

### Investigation of MMP-associated genes in CD and UC *via* single-cell sequencing

**Figure 11:**
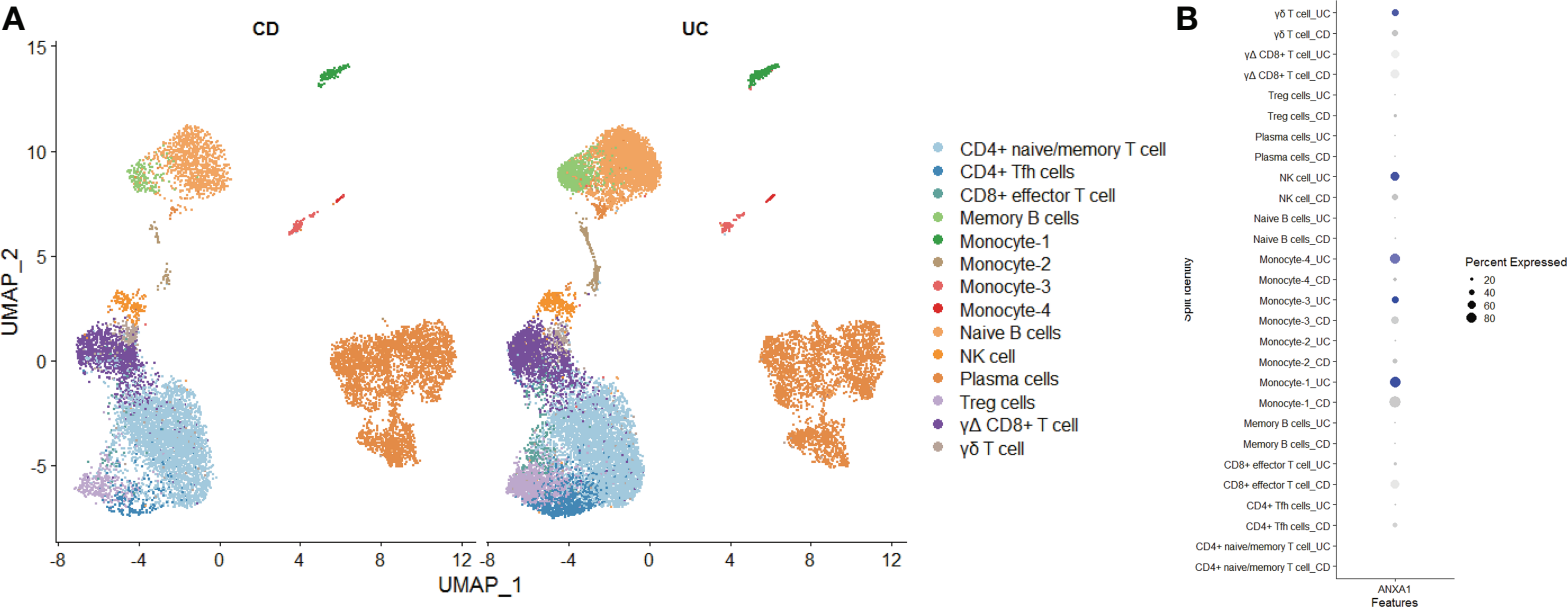
Exploring the Expression of MMP-Associated Genes by Single-cell Sequencing **(A)** Intestinal mucosa samples from 14 IBD patients (7 UC and 7 CD) were integrated through the Harmony method and are displayed in a UMAP reduction diagram. **(B)** ANXA1 expression in different cell clusters.

Finally, we used single-cell sequencing technology to explore the expression of MMP-associated genes in IBD. Using GSE125527, we analyzed a total of 14 samples, including 7 UC patients and 7 CD patients. Through harmony integration technology, we reduced the batch differences between the different samples to better analyze gene expression (**Figure 11A**). Due to the limitation of single-cell sequencing technology in sequencing depth, most MMP-associated genes were not detected, except for ANXA1. The results showed that the expression of ANXA1 in UC was higher than that in CD, and the cells with a high expression of ANXA1 were mainly monocytes, NK cells and γδ T cells (**Figure 11B**).

## Discussion

UC and CD are subtypes of inflammatory bowel disease and are autoimmune diseases influenced by multiple complex factors, including the environment, genetic factors, and the gut microbiota. The specific pathogenesis underlying IBD remains unclear despite extensive research investigating the disease over many years. The clinical treatment strategies used for UC and CD patients are often different. For example, it is recommended that aminosalicylates should be used as a first-line approach for treating and maintaining remission in UC, but they play a much smaller role in the management of CD (6, 7, 32, 33). In contrast, methotrexate has shown a higher rate of response in CD patients than in UC patients (34–36). However, there remains a large number of IBD patients who are difficult to identify in clinical practice, indicating that it is important to improve the discrimination of the two different subtypes, especially since the results of differential diagnosis affect clinical management (21).

In this study, we systematically collected and integrated published microarray data obtained from CD and UC patients. The first challenge we faced was determining the best strategy for integrating these data to best identify the DEGs. Importantly, the choice of integration method has an impact on the analysis results. If bias is introduced during the integration of the data, the results and their interpretation are inevitably affected. An RRA analysis was adopted to integrate the data to identify the DEGs. RRA is based on a comparison of actual data with a null model that assumes a random order of input lists; then, a P value is assigned to the difference in the levels of each gene in the aggregated list that describes how much better a gene ranked than expected. As an algorithm that is both computationally efficient and statistically stable, RRA has obvious advantages. First, due to scoring based on the order of gene expression, this method is very robust and can accommodate the variable gene content that results from the use of different microarray platforms. Second, even if a gene is not indicated in one platform, it is not eliminated due to the combination of multiple datasets to prevent losing information regarding important genes. This conjecture can also be confirmed from the analysis results. For example, in the GSE75214 dataset, we identified many differentially expressed genes, while relatively few DEGs were identified in the other three datasets, proving the heterogeneity of the data obtained from different central sources (**Figures 2A-D**).

However, RRA also has shortcomings. For example, when the final results from different datasets are summarized by an RRA, the results obtained from large sample sizes and small sample sizes are given the same weight, which may lead to issues in the representativeness of the final results. Therefore, after conducting the RRA, we used another method of data consolidation. Datasets from the same gene platform were merged with the removal of batch variance. Then, the DEGs were identified in the Combined Datasets and the GSE75214 dataset, and the final DEGs were identified by considering the intersection of the DEGs between the two IBD cohorts. The gene enrichment analysis showed that the pathways enriched by the DEGs identified by the two integration methods were similar, indicating the robustness of the DEG screening results. Another noteworthy finding was that the IL-17 signaling pathway was the most significant pathway enriched in UC. This result was similar to the results of a recently published single-cell sequencing study that reported that IL17A+ T cells are mainly enriched in UC (37).

The DEGs identified by the two different filtering strategies were further used to generate PPI networks, and pivotal gene modules were identified through MCODE. Interestingly, the results from both sets of DEGs indicated that the MMP-associated module was the most important gene set differing between CD and UC patients, providing credible evidence that the MMP-associated module serves as the main functional group associated with the difference between CD and UC.

In future studies, we plan to establish a differential diagnosis model based on the MMP-associated module to assist in the diagnosis of the CD and UC subtypes in IBD patients. To the best of our knowledge, this study offers the first publicly reported mRNA model for the differential diagnosis of IBD, although some studies have reported the role of miRNAs in the differential diagnosis of IBD (20). In this study, combined datasets were used as the training group, and the GSE75214 dataset was used as the testing group to generate Model_1 *via* a LASSO logistic regression. From the results of the model evaluation, Model_1 showed good discrimination in both the training group and the testing group. Further studies using a calibration analysis and DCA showed that Model_ 1 exhibited good performance in the training group but failed in the testing group. We believe the reason is that the data obtained from the training set and the testing set were collected from different microarray platforms, resulting in large batch differences between the two IBD cohorts. Although Model_ 1 based on the training set could better distinguish the data obtained from other platforms, it was difficult to meet higher accuracy requirements. This challenge is an inherent defect of diagnostic models obtained based on microarray technology, which also limits the application of such diagnostic models; thus, a diagnostic model can only be applied to clinical data collected from the same microarray platform in real clinical diagnosis.

Based on the above, another model named Model_ 2 was generated. In the new model, we omitted the specific expression values of the genes and converted them into binary variables; thus, we did not need to consider the problem of batch differences. Subsequently, we used the Combined Datasets as the training group and the GSE75214 dataset as the testing group to generate a LASSO logistic regression model. Interestingly, although the continuous variables in the matrix were transformed into binary variables, the genes identified using Model_2 were generally the same as those identified using Model_1 (ANXA1, MMP10, MMP1 and CXCL13 in Model_1 *vs.* ANXA1, MMP1 CXCL13 and CXCL1 in Model_2). In the subsequent model evaluation, we found that Model_2 showed good discrimination in both the training set and the testing set. More importantly, Model_2 also performed well in both the training group and testing group based on the results of the calibration analysis and DCA. Notably, because Model_2 only included 4 genes and the levels of each gene were represented by a binary variable, the actual values obtained using Model_2 included only 16 different values at most, further indicating the reliability of the analysis based on the included genes. Most importantly, the newly included cohort GSE179285 contains CD samples from colon tissue. In the newly added validation results, we performed a model validation using IBD cases containing colonic/ileal CD and IBD cohorts containing colonic CD only, and the validation results were consistent with our expected results. In the subsequent experiment, we explored the diagnostic value of the MMP-associated genes. The results showed that the use of a single-gene diagnostic strategy performed better, and these results were further confirmed in clinical samples.

Matrix metalloproteinases (MMPs) constitute a group of zinc-dependent neutral peptidases that can degrade all components of the extracellular matrix (ECM) and are associated with extensive mucosal degradation and tissue remodeling, which ultimately favor the development of ulcers, fistulae and strictures (38). According to their primary substrate, MMPs can be divided into various subclasses. The genes identified in this research included stromelysins (MMP-3, MMP-7, and MMP-10) and collagenases (MMP-1). To date, sufficient evidence suggests that IBD-related mucosal inflammation is associated with an enhanced induction of several MMPs. For example, a series of pioneering studies documented the abundant expression of MMP-1 (39–42) and MMP-3 (39, 43–46) RNA in gastrointestinal tissue surrounding ulcers, including those present in the gut of IBD patients. Subsequent research has further proven that MMP-7 (47–51) and MMP-10 (47, 48, 52–54) RNA expression levels are increased in the inflamed tissue of UC patients. Considering that adequate functional studies support the involvement of MMPs in IBD-related mucosal degradation, several inhibitors of MMPs have been developed and used to attenuate gut inflammation in animal models of IBD (55–57), and 3 clinical trials investigating MMP inhibitors have been performed in the context of IBD treatment (58–61). However, our research indicates that the MMP-associated module was also the main differential gene set between CD and UC. CIBERSORT showed that MMP-associated genes were closely associated with unique immune characteristics in UC, including higher levels of neutrophils and humoral immune cells (naive B cells and follicular helper T cells) and lower levels of Treg cells than in CD patients.

Regarding the other genes included in the models, chemokine C-X-C motif ligand-1 (CXCL1) is widely known as a strong neutrophil chemoattractant that participates in inflammation in multiple tissues. MMP3-CXCL1 (62) and MMP7-CXCL1 (63) often function as partners in neutrophil activation or as biomarkers of the dysplasia-carcinoma transition in sporadic colorectal cancer. The chemokine CXC ligand 13 (CXCL13), also named B-cell-attracting chemokine-1 (BAC-1) or B-lymphocyte-chemoattractant (BLC), is a CXC subtype member of the chemokine superfamily that serves as an inflammatory mediator linked to B lymphocyte activity and lymphoid-neogenesis (64). Recently, UC was characterized as exhibiting a plasmablast-skewed humoral response associated with disease activity, and a subset of intestinal CXCL13-expressing TFH-like T peripheral helper cells was identified to be associated with the pathogenic B-cell response (65). Another molecule that deserves attention is annexin A1 (ANXA1) because it played an important role in both models. According to early studies of UC, one of the characteristics of an active episode of US is the intense mucosal infiltration of leukocytes, and the proresolution mediator ANXA1 exerts counterregulatory effects on leukocyte recruitment and exhibits elevated levels in sera isolated from active IBD patients (66). Further studies reported that ANXA1 was packaged in extracellular vesicles (EVs) derived from IECs (67), indicating that an analysis of the increasing levels of ANXA1 in IEC-derived EVs may become a specific diagnostic approach for IBD clinical diagnosis (68–71). By conducting a single-cell sequencing analysis, we searched for ANXA1 expression traces more precisely. The results showed that ANXA1 is mainly expressed in monocytes, including macrophages and dendritic cells. More studies are needed to explore the biological functions of ANXA1 in IBD.

The advantages of this study include the following: 1) to the best of our knowledge, this study is the most recently published study using CD and UC microarray data with the largest sample size and the first to offer an mRNA-based model for differential diagnosis; 2) this study strictly followed the guidelines of the TRIPOD and performed a comprehensive evaluation of the generated model; and 3) the generated Model_2 overcame the problem of batch differences and had good clinical applicability. However, this study also had some shortcomings. The data used during the model establishment were obtained from a public database, and the results need to be verified using a larger amount of clinical data. Although we collected a certain number of clinical samples for IHC testing in our article, the number was small. In particular, due to the lack of examination of difficult cases, it is not possible to assess whether the model can be used as an aid in the diagnosis of difficult IBD cases.

## Conclusion

Although both CD and UC are types of IBD and exhibit similar clinical symptoms, there are many differences in the immune landscape. This finding can explain to some extent why CD and UC patients exhibit different responses after receiving the same treatment. In recent years, single-cell sequencing has been used to describe the immune landscape of CD and UC patients, which has enabled a more accurate identification of immune cell differences (37). However, due to the heterogeneity and complexity of IBD, data analyses based on larger sample sizes and multicenter data through high-throughput microarray remains important.

Our study revealed that the MMP-associated module is an important differential functional set in CD and UC, and based on this, we established two models to assist in the differential diagnosis of CD and UC in the clinic. The comprehensive model evaluation demonstrated that the model based on the MMP-associated module had good application value. Subsequent in-depth research investigating how MMPs are involved in the development of different subtypes of IBD is necessary.

## Data availability statement

The original contributions presented in the study are included in the article/**Supplementary Material**. Further inquiries can be directed to the corresponding authors.

## Ethics statement

The studies involving human participants were reviewed and approved by the ethics committee of the second hospital of Hebei Medical University. The ethics committee waived the requirement of written informed consent for participation.

## Author contributions

JD collected the papers, analyzed the data, and drafted the manuscript. NZ and L-pL analyzed the data. PM and Y-yaZ reviewed the data and conclusions. J-bX and X-pZ contributed to manuscript writing. Z-aC and Y-yuZ presented the idea for this manuscript, supported the funding, analyzed the conclusions, and drafted and revised the manuscript. All authors contributed to the article and approved the submitted version.

## Funding

This study was funded by the National Natural Science Foundation of China (Grant No. 81902055, Grant No. 82200738) and the Medical Science Research Projects of Hebei Province (Grant No. 20220990).

## Conflict of interest

The authors declare that the research was conducted in the absence of any commercial or financial relationships that could be construed as a potential conflict of interest.

## Publisher’s note

All claims expressed in this article are solely those of the authors and do not necessarily represent those of their affiliated organizations, or those of the publisher, the editors and the reviewers. Any product that may be evaluated in this article, or claim that may be made by its manufacturer, is not guaranteed or endorsed by the publisher.

## References

[1] de Souza HS Fiocchi C. Immunopathogenesis of IBD: Current state of the art. Nat Rev Gastroenterol Hepatol (2016) 13(1):13–27. doi: 10.1038/nrgastro.2015.186 26627550

[2] Bouma G Strober W. The immunological and genetic basis of inflammatory bowel disease. Nat Rev Immunol (2003) 3(7):521–33. doi: 10.1038/nri1132 12876555

[3] Hendrickson BA Gokhale R Cho JH. Clinical aspects and pathophysiology of inflammatory bowel disease. Clin Microbiol Rev (2002) 15(1):79–94. doi: 10.1128/CMR.15.1.79-94.2002 11781268 PMC118061

[4] Seyedian SS Nokhostin F Malamir MD. A review of the diagnosis, prevention, and treatment methods of inflammatory bowel disease. J Med Life (2019) 12(2):113–22. doi: 10.25122/jml-2018-0075 PMC6685307 31406511

[5] Feakins RM. Ulcerative colitis or crohn's disease? pitfalls and problems. Histopathology (2014) 64(3):317–35. doi: 10.1111/his.12263 24266813

[6] Dignass A Lindsay JO Sturm A Windsor A Colombel JF Allez M. Second european evidence-based consensus on the diagnosis and management of ulcerative colitis part 2: Current management. J Crohns Colitis (2012) 6(10):991–1030. doi: 10.1016/j.crohns.2012.09.002 23040451

[7] Dignass A Van Assche G Lindsay JO Lémann M Söderholm J Colombel JF. The second european evidence-based consensus on the diagnosis and management of crohn's disease: Current management. J Crohns Colitis (2010) 4(1):28–62. doi: 10.1016/j.crohns.2009.12.002 21122489

[8] Tun GS Cripps S Lobo AJ. Crohn's disease: Management in adults, children and young people - concise guidance. Clin Med (Lond) (2018) 18(3):231–6. doi: 10.7861/clinmedicine.18-3-231 PMC6334085 29858433

[9] Kornbluth A Sachar DB. Ulcerative colitis practice guidelines in adults (update): American college of gastroenterology, practice parameters committee. Am J Gastroenterol (2004) 99(7):1371–85. doi: 10.1111/j.1572-0241.2004.40036.x 15233681

[10] Magro F Langner C Driessen A Ensari A Geboes K Mantzaris GJ. European consensus on the histopathology of inflammatory bowel disease. J Crohns Colitis (2013) 7(10):827–51. doi: 10.1016/j.crohns.2013.06.001 23870728

[11] Annese V Daperno M Rutter MD Amiot A Bossuyt P East J. European evidence based consensus for endoscopy in inflammatory bowel disease. J Crohns Colitis (2013) 7(12):982–1018. doi: 10.1016/j.crohns.2013.09.016 24184171

[12] Meucci G Bortoli A Riccioli FA Girelli CM Radaelli F Rivolta R. Frequency and clinical evolution of indeterminate colitis: A retrospective multi-centre study in northern italy. GSMII (Gruppo di studio per le malattie infiammatorie intestinali). Eur J Gastroenterol Hepatol (1999) 11(8):909–13. 10514127

[13] Nuij VJ Zelinkova Z Rijk MC Beukers R Ouwendijk RJ Quispel R. Phenotype of inflammatory bowel disease at diagnosis in the netherlands: a population-based inception cohort study (the delta cohort). Inflammation Bowel Dis (2013) 19(10):2215–22. doi: 10.1097/MIB.0b013e3182961626 23835444

[14] Burisch J Pedersen N Čuković-Čavka S Brinar M Kaimakliotis I Duricova D. East-west gradient in the incidence of inflammatory bowel disease in europe: the ECCO-EpiCom inception cohort. Gut (2014) 63(4):588–97. doi: 10.1136/gutjnl-2013-304636 23604131

[15] Melmed GY Elashoff R Chen GC Nastaskin I Papadakis KA Vasiliauskas EA. Predicting a change in diagnosis from ulcerative colitis to crohn's disease: A nested, case-control study. Clin Gastroenterol Hepatol (2007) 5(5):602–8. doi: 10.1016/j.cgh.2007.02.015 17478347

[16] Myren J Bouchier IA Watkinson G Softley A Clamp SE de Dombal FT. The OMGE multinational inflammatory bowel disease survey 1976-1986. A further Rep 3175 cases. Scand J Gastroenterol Suppl (1988) 144:11–9. 3165550

[17] Abraham BP Mehta S El-Serag HB. Natural history of pediatric-onset inflammatory bowel disease: A systematic review. J Clin Gastroenterol (2012) 46(7):581–9. doi: 10.1097/MCG.0b013e318247c32f PMC3972042 22772738

[18] Marcello PW Schoetz DJ Jr Roberts PL Murray JJ Coller JA Rusin LC. Evolutionary changes in the pathologic diagnosis after the ileoanal pouch procedure. Dis Colon Rectum (1997) 40(3):263–9. doi: 10.1007/BF02050413 9118738

[19] Henriksen M Jahnsen J Lygren I Sauar J Schulz T Stray N. Change of diagnosis during the first five years after onset of inflammatory bowel disease: Results of a prospective follow-up study (the IBSEN study). Scand J Gastroenterol (2006) 41(9):1037–43. doi: 10.1080/00365520600554527 16938716

[20] James JP Riis LB Malham M Høgdall E Langholz E Nielsen BS. MicroRNA biomarkers in IBD-differential diagnosis and prediction of colitis-associated cancer. Int J Mol Sci (2020) 21(21). doi: 10.3390/ijms21217893 PMC7660644 33114313

[21] Tontini GE Vecchi M Pastorelli L Neurath MF Neumann H. Differential diagnosis in inflammatory bowel disease colitis: State of the art and future perspectives. World J Gastroenterol (2015) 21(1):21–46. doi: 10.3748/wjg.v21.i1.21 25574078 PMC4284336

[22] Derkacz A Olczyk P Olczyk K Komosinska-Vassev K. The role of extracellular matrix components in inflammatory bowel diseases. J Clin Med (2021) 10(5). doi: 10.3390/jcm10051122 PMC7962650 33800267

[23] Yu G Wang LG Han Y He QY. clusterProfiler: An r package for comparing biological themes among gene clusters. OMICS (2012) 16(5):284–7. doi: 10.1089/omi.2011.0118 PMC3339379 22455463

[24] Smoot ME Ono K Ruscheinski J Wang PL Ideker T. Cytoscape 2.8: new features for data integration and network visualization. Bioinformatics (2011) 27(3):431–2. doi: 10.1093/bioinformatics/btq675 PMC3031041 21149340

[25] Moons KG Altman DG Reitsma JB Ioannidis JP Macaskill P Steyerberg EW. Transparent reporting of a multivariable prediction model for individual prognosis or diagnosis (TRIPOD): Explanation and elaboration. Ann Intern Med (2015) 162(1):W1–73. doi: 10.7326/M14-0698 25560730

[26] Zhang H Luo YB Wu W Zhang L Wang Z Dai Z. The molecular feature of macrophages in tumor immune microenvironment of glioma patients. Comput Struct Biotechnol J (2021) 19:4603–18. doi: 10.1016/j.csbj.2021.08.019 PMC8383063 34471502

[27] Varghese F Bukhari AB Malhotra R De A. IHC profiler: an open source plugin for the quantitative evaluation and automated scoring of immunohistochemistry images of human tissue samples. PloS One (2014) 9(5):e96801. doi: 10.1371/journal.pone.0096801 24802416 PMC4011881

[28] Vancamelbeke M Vanuytsel T Farré R Verstockt S Ferrante M Van Assche G. Genetic and transcriptomic bases of intestinal epithelial barrier dysfunction in inflammatory bowel disease. Inflammation Bowel Dis (2017) 23(10):1718–29. doi: 10.1097/MIB.0000000000001246 PMC6461205 28885228

[29] Kugathasan S Baldassano RN Bradfield JP Sleiman PM Imielinski M Guthery SL. Loci on 20q13 and 21q22 are associated with pediatric-onset inflammatory bowel disease. Nat Genet (2008) 40(10):1211–5. doi: 10.1038/ng.203 PMC2770437 18758464

[30] Montero-Meléndez T Llor X García-Planella E Perretti M Suárez A. Identification of novel predictor classifiers for inflammatory bowel disease by gene expression profiling. PloS One (2013) 8(10):e76235. doi: 10.1371/journal.pone.0076235 24155895 PMC3796518

[31] Carey R Jurickova I Ballard E Bonkowski E Han X Xu H. Activation of an IL-6:STAT3-dependent transcriptome in pediatric-onset inflammatory bowel disease. Inflammation Bowel Dis (2008) 14(4):446–57. doi: 10.1002/ibd.20342 PMC2581837 18069684

[32] Lim WC Hanauer S. Aminosalicylates for induction of remission or response in crohn's disease. Cochrane Database Syst Rev (2010) 12):CD008870. doi: 10.1002/14651858.CD008870 21154400

[33] Gisbert JP Chaparro M Gomollón F. Common misconceptions about 5-aminosalicylates and thiopurines in inflammatory bowel disease. World J Gastroenterol (2011) 17(30):3467–78. doi: 10.3748/wjg.v17.i30.3467 PMC3163244 21941413

[34] Feagan BG Fedorak RN Irvine EJ Wild G Sutherland L Steinhart AH. A comparison of methotrexate with placebo for the maintenance of remission in crohn's disease. North Am Crohn's Study Group Investigators. N Engl J Med (2000) 342(22):1627–32. doi: 10.1056/NEJM200006013422202 10833208

[35] Saibeni S Bollani S Losco A Michielan A Sostegni R Devani M. The use of methotrexate for treatment of inflammatory bowel disease in clinical practice. Dig Liver Dis (2012) 44(2):123–7. doi: 10.1016/j.dld.2011.09.015 22051323

[36] Laharie D Reffet A Belleannée G Chabrun E Subtil C Razaire S. Mucosal healing with methotrexate in crohn's disease: A prospective comparative study with azathioprine and infliximab. Aliment Pharmacol Ther (2011) 33(6):714–21. doi: 10.1111/j.1365-2036.2010.04569.x 21235604

[37] Mitsialis V Wall S Liu P Ordovas-Montanes J Parmet T Vukovic M. Single-cell analyses of colon and blood reveal distinct immune cell signatures of ulcerative colitis and crohn's disease. Gastroenterology (2020) 159(2):591–608.e10. doi: 10.1053/j.gastro.2020.04.074 32428507 PMC8166295

[38] Marônek M Marafini I Gardlík R Link R Troncone E Monteleone G. Metalloproteinases in inflammatory bowel diseases. J Inflammation Res (2021) 14:1029–41. doi: 10.2147/JIR.S288280 PMC8001665 33790618

[39] Pender SL Tickle SP Docherty AJ Howie D Wathen NC MacDonald TT. A major role for matrix metalloproteinases in t cell injury in the gut. J Immunol (1997) 158(4):1582–90. 9029093

[40] Saarialho-Kere UK Vaalamo M Puolakkainen P Airola K Parks WC Karjalainen-Lindsberg ML. Enhanced expression of matrilysin, collagenase, and stromelysin-1 in gastrointestinal ulcers. Am J Pathol (1996) 148(2):519–26. PMC1861683 8579114

[41] Manuc M Ionescu EM Milanesi E Dobre M Tieranu I Manuc TE. Molecular signature of persistent histological inflammation in ulcerative colitis with mucosal healing. J Gastrointestin Liver Dis (2020) 29(2):159–66. doi: 10.15403/jgld-576 32530982

[42] Arihiro S Ohtani H Hiwatashi N Torii A Sorsa T Nagura H. Vascular smooth muscle cells and pericytes express MMP-1, MMP-9, TIMP-1 and type i procollagen in inflammatory bowel disease. Histopathology (2001) 39(1):50–9. doi: 10.1046/j.1365-2559.2001.01142.x 11454044

[43] Salmela MT MacDonald TT Black D Irvine B Zhuma T Saarialho-Kere U. Upregulation of matrix metalloproteinases in a model of t cell mediated tissue injury in the gut: analysis by gene array and *in situ* hybridisation. Gut (2002) 51(4):540–7. doi: 10.1136/gut.51.4.540 PMC1773375 12235077

[44] Kirkegaard T Hansen A Bruun E Brynskov J. Expression and localisation of matrix metalloproteinases and their natural inhibitors in fistulae of patients with crohn's disease. Gut (2004) 53(5):701–9. doi: 10.1136/gut.2003.017442 PMC1774029 15082589

[45] Louis E Ribbens C Godon A Franchimont D De Groote D Hardy N. Increased production of matrix metalloproteinase-3 and tissue inhibitor of metalloproteinase-1 by inflamed mucosa in inflammatory bowel disease. Clin Exp Immunol (2000) 120(2):241–6. doi: 10.1046/j.1365-2249.2000.01227.x PMC1905637 10792371

[46] Heuschkel RB MacDonald TT Monteleone G Bajaj-Elliott M Smith JA Pender SL. Imbalance of stromelysin-1 and TIMP-1 in the mucosal lesions of children with inflammatory bowel disease. Gut (2000) 47(1):57–62. doi: 10.1136/gut.47.1.57 10861265 PMC1727949

[47] Rath T Roderfeld M Graf J Wagner S Vehr AK Dietrich C. Enhanced expression of MMP-7 and MMP-13 in inflammatory bowel disease: a precancerous potential. Inflammation Bowel Dis (2006) 12(11):1025–35. doi: 10.1097/01.mib.0000234133.97594.04 17075343

[48] Jimbo K Ohtsuka Y Kojima Y Hosoi K Ohbayashi N Ikuse T. Increased expression of CXCR3 axis components and matrix metalloproteinase in pediatric inflammatory bowel disease patients. Pediatr Int (2014) 56(6):873–83. doi: 10.1111/ped.12362 24750209

[49] Matsuno K Adachi Y Yamamoto H Goto A Arimura Y Endo T. The expression of matrix metalloproteinase matrilysin indicates the degree of inflammation in ulcerative colitis. J Gastroenterol (2003) 38(4):348–54. doi: 10.1007/s005350300062 12743774

[50] Rath T Roderfeld M Halwe JM Tschuschner A Roeb E Graf J. Cellular sources of MMP-7, MMP-13 and MMP-28 in ulcerative colitis. Scand J Gastroenterol (2010) 45(10):1186–96. doi: 10.3109/00365521.2010.499961 20568971

[51] Newell KJ Matrisian LM Driman DK. Matrilysin (matrix metalloproteinase-7) expression in ulcerative colitis-related tumorigenesis. Mol Carcinog (2002) 34(2):59–63. doi: 10.1002/mc.10049 12112311

[52] Dobre M Milanesi E Mănuc TE Arsene DE Ţieranu CG Maj C. Differential intestinal mucosa transcriptomic biomarkers for crohn's disease and ulcerative colitis. J Immunol Res (2018) 2018:9208274. doi: 10.1155/2018/9208274 30417021 PMC6207860

[53] Salmela MT Pender SL Karjalainen-Lindsberg ML Puolakkainen P Macdonald TT Saarialho-Kere U. Collagenase-1 (MMP-1), matrilysin-1 (MMP-7), and stromelysin-2 (MMP-10) are expressed by migrating enterocytes during intestinal wound healing. Scand J Gastroenterol (2004) 39(11):1095–104. doi: 10.1080/00365520410003470 15545168

[54] Mäkitalo L Kolho KL Karikoski R Anthoni H Saarialho-Kere U. Expression profiles of matrix metalloproteinases and their inhibitors in colonic inflammation related to pediatric inflammatory bowel disease. Scand J Gastroenterol (2010) 45(7-8):862–71. doi: 10.3109/00365520903583863 20367198

[55] O'Sullivan S Wang J Radomski MW Gilmer JF Medina C. Novel barbiturate-nitrate compounds inhibit the upregulation of matrix metalloproteinase-9 gene expression in intestinal inflammation through a cGMP-mediated pathway. Biomolecules (2020) 10(5). doi: 10.3390/biom10050808 PMC7277209 32466182

[56] Sykes AP Bhogal R Brampton C Chander C Whelan C Parsons ME. The effect of an inhibitor of matrix metalloproteinases on colonic inflammation in a trinitrobenzenesulphonic acid rat model of inflammatory bowel disease. Aliment Pharmacol Ther (1999) 13(11):1535–42. doi: 10.1046/j.1365-2036.1999.00633.x 10571613

[57] Di Sebastiano P di Mola FF Artese L Rossi C Mascetta G Pernthaler H. Beneficial effects of batimastat (BB-94), a matrix metalloproteinase inhibitor, in rat experimental colitis. Digestion (2001) 63(4):234–9. doi: 10.1159/000051895 11435723

[58] Schreiber S Siegel CA Friedenberg KA Younes ZH Seidler U Bhandari BR. A phase 2, randomized, placebo-controlled study evaluating matrix metalloproteinase-9 inhibitor, andecaliximab, in patients with moderately to severely active crohn's disease. J Crohns Colitis (2018) 12(9):1014–20. doi: 10.1093/ecco-jcc/jjy070 PMC6113705 29846530

[59] Sandborn WJ Bhandari BR Fogel R Onken J Yen E Zhao X. Randomised clinical trial: A phase 1, dose-ranging study of the anti-matrix metalloproteinase-9 monoclonal antibody GS-5745 versus placebo for ulcerative colitis. Aliment Pharmacol Ther (2016) 44(2):157–69. doi: 10.1111/apt.13653 PMC5089609 27218676

[60] Sandborn WJ Bhandari BR Randall C Younes ZH Romanczyk T Xin Y. Andecaliximab [Anti-matrix metalloproteinase-9] induction therapy for ulcerative colitis: A randomised, double-blind, placebo-controlled, phase 2/3 study in patients with moderate to severe disease. J Crohns Colitis (2018) 12(9):1021–9. doi: 10.1093/ecco-jcc/jjy049 PMC6113706 29767728

[61] Appleby TC Greenstein AE Hung M Liclican A Velasquez M Villaseñor AG. Biochemical characterization and structure determination of a potent, selective antibody inhibitor of human MMP9. J Biol Chem (2017) 292(16):6810–20. doi: 10.1074/jbc.M116.760579 PMC5399127 28235803

[62] Sipos F Germann TM Wichmann B Galamb O Spisák S Krenács T. MMP3 and CXCL1 are potent stromal protein markers of dysplasia-carcinoma transition in sporadic colorectal cancer. Eur J Cancer Prev (2014) 23(5):336–43. doi: 10.1097/CEJ.0000000000000058 24999605

[63] Gill SE Nadler ST Li Q Frevert CW Park PW Chen P. Shedding of syndecan-1/CXCL1 complexes by matrix metalloproteinase 7 functions as an epithelial checkpoint of neutrophil activation. Am J Respir Cell Mol Biol (2016) 55(2):243–51. doi: 10.1165/rcmb.2015-0193OC PMC4979362 26934670

[64] Rymarz A Mosakowska M Niemczyk S. The significance of metalloproteinase 3 (MMP-3), chemokine CXC ligand 13 (CXCL-13) and complement component C5a in different stages of ANCA associated vasculitis. Sci Rep (2021) 11(1):5132. doi: 10.1038/s41598-021-84662-3 33664330 PMC7933137

[65] Uzzan M Martin JC Mesin L Livanos AE Castro-Dopico T Huang R. Ulcerative colitis is characterized by a plasmablast-skewed humoral response associated with disease activity. Nat Med (2022) 28(4):766–79. doi: 10.1038/s41591-022-01680-y PMC9107072 35190725

[66] Vong L Ferraz JG Dufton N Panaccione R Beck PL Sherman PM. Up-regulation of annexin-A1 and lipoxin A(4) in individuals with ulcerative colitis may promote mucosal homeostasis. PloS One (2012) 7(6):e39244. doi: 10.1371/journal.pone.0039244 22723974 PMC3377644

[67] Leoni G Neumann PA Kamaly N Quiros M Nishio H Jones HR. Annexin A1-containing extracellular vesicles and polymeric nanoparticles promote epithelial wound repair. J Clin Invest (2015) 125(3):1215–27. doi: 10.1172/JCI76693 PMC4362251 25664854

[68] Shen Q Huang Z Yao J Jin Y. Extracellular vesicles-mediated interaction within intestinal microenvironment in inflammatory bowel disease. J Adv Res (2022) 37:221–33. doi: 10.1016/j.jare.2021.07.002 PMC9039646 35499059

[69] Leoni G Alam A Neumann PA Lambeth JD Cheng G McCoy J. Annexin A1, formyl peptide receptor, and NOX1 orchestrate epithelial repair. J Clin Invest (2013) 123(1):443–54. doi: 10.1172/JCI65831 PMC3533303 23241962

[70] Martin GR Perretti M Flower RJ Wallace JL. Annexin-1 modulates repair of gastric mucosal injury. Am J Physiol Gastrointest Liver Physiol (2008) 294(3):G764–9. doi: 10.1152/ajpgi.00531.2007 18202108

[71] Perretti M D'Acquisto F. Annexin A1 and glucocorticoids as effectors of the resolution of inflammation. Nat Rev Immunol (2009) 9(1):62–70. doi: 10.1038/nri2470 19104500

