# Supplementary material for: Integrated analysis of multiple microarray studies to establish differential diagnostic models of Crohn’s disease and ulcerative colitis based on a metalloproteinase-associated module
Source: Front Immunol. 2022 Nov 21;13:1022850. doi: 10.3389/fimmu.2022.1022850 (PMC9720321; doi:10.3389/fimmu.2022.1022850)
Supplement: Supplementary file 1 [file DataSheet_1.docx]

**
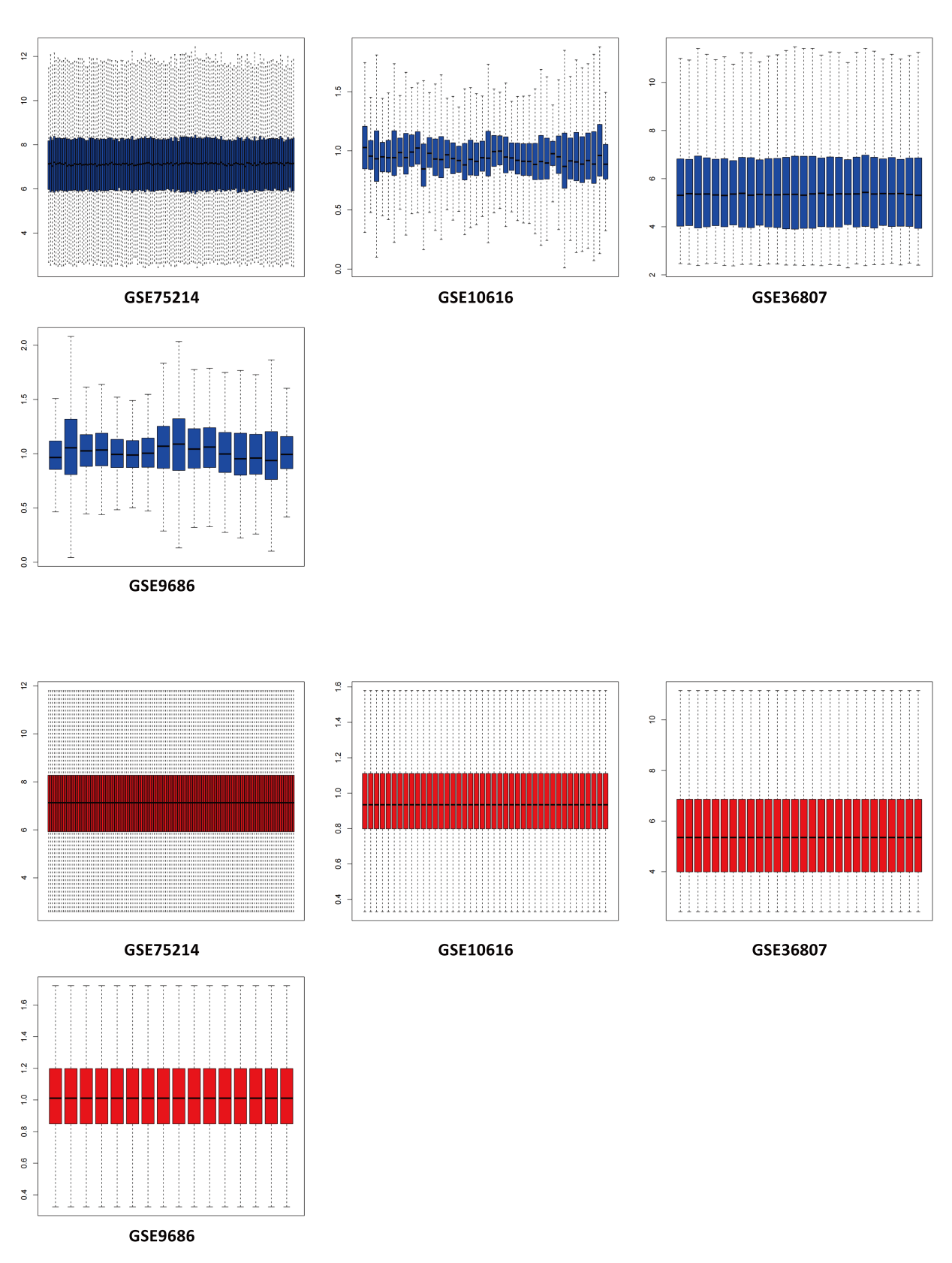
**

**Supplementary Figure 1**：Standardization of gene expression. Standardization of gene expression in GSE75214, GSE10616, GSE36807, and GSE9686 datasets. The blue bar represents the data before normalization, and the red bar represents the data after normalization.


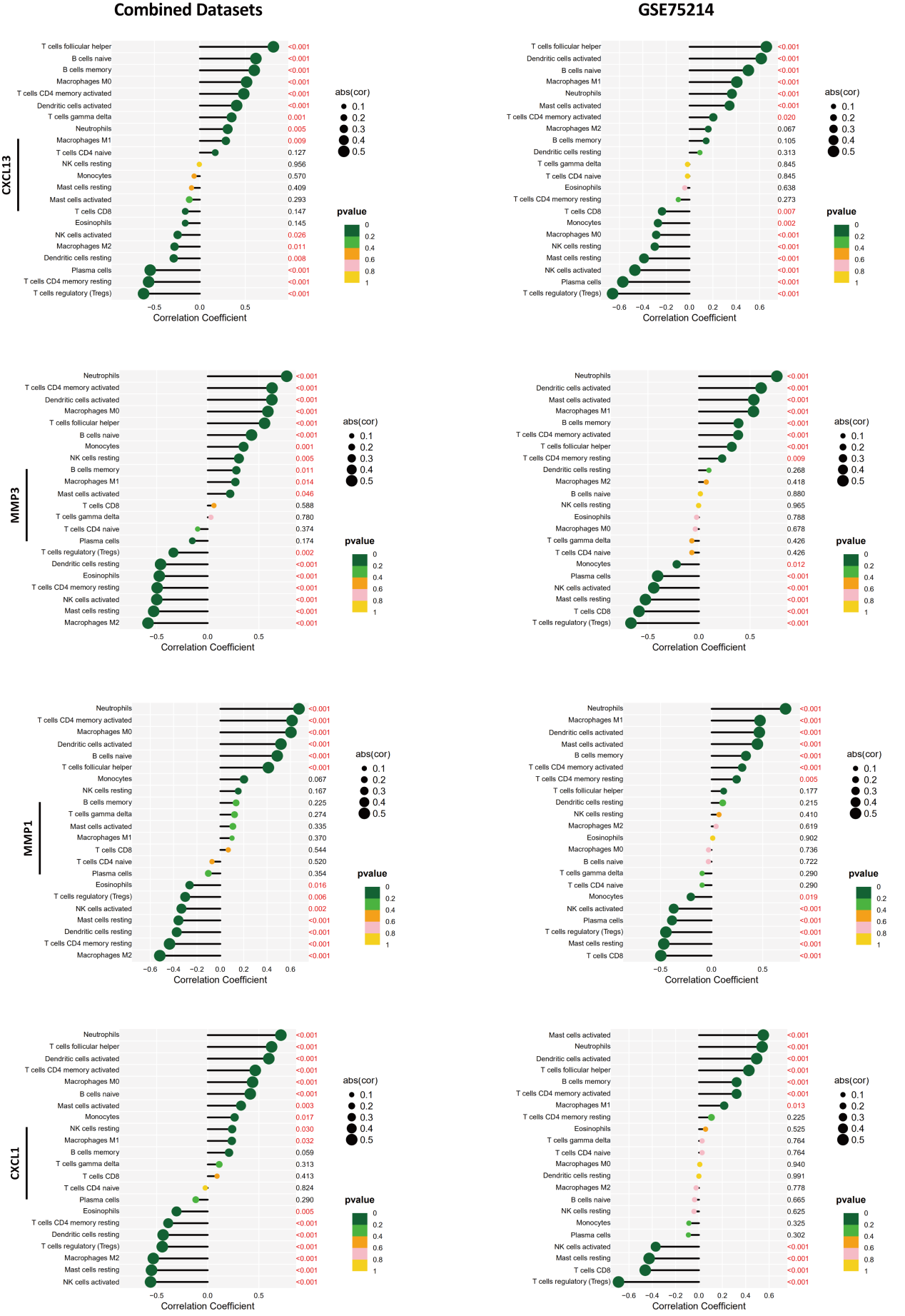


**Supplementary Figure 2：**Correlations between the MMP-associated genes and immune cells were further examined by Spearman correlation analysis in the Combined Datasets and GSE75214.

**
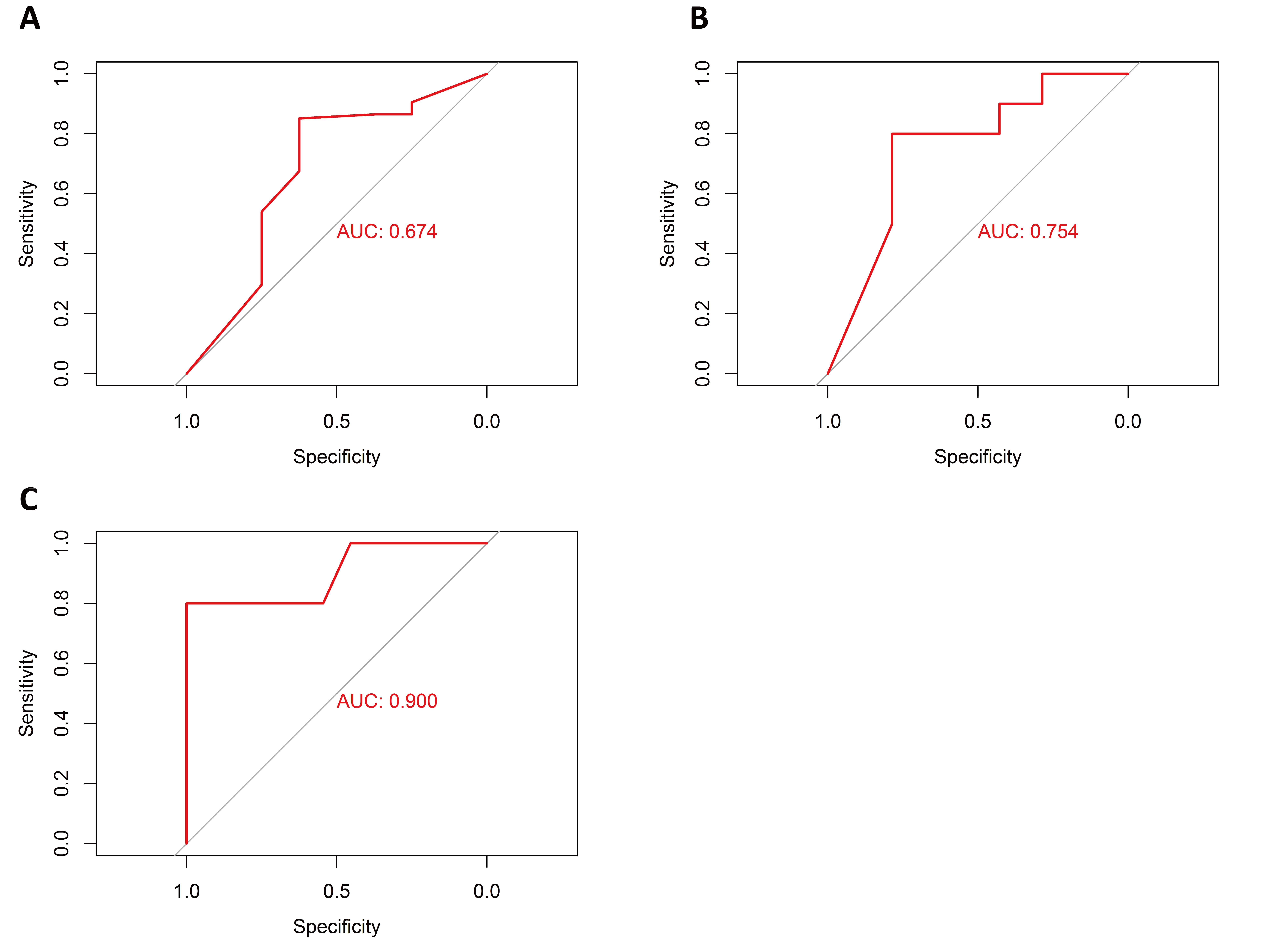
**

**Supplementary Figure 3: Verification of the Effect of Model_2 in the Colonic Dominant CD and UC Cohort.** The ROC curve were illustrated to test the effect of Model 2 in GSE75214 (A), GSE10616 (B), and GSE9686 (C). Since only CD samples from colonic tissue and UC are compared here, CD/UC=8/74 for the sample size in the GSE75214, CD/UC=14/10 for the sample size in the GSE10616, and CD/UC=11/5 for the sample size in the GSE9686.
